# Luteolin Suppresses Cancer Cell Proliferation by Targeting Vaccinia-Related Kinase 1

**DOI:** 10.1371/journal.pone.0109655

**Published:** 2014-10-13

**Authors:** Ye Seul Kim, Seong-Hoon Kim, Joon Shin, Amaravadhi Harikishore, Jong-Kwan Lim, Youngseob Jung, Ha-Na Lyu, Nam-In Baek, Kwan Yong Choi, Ho Sup Yoon, Kyong-Tai Kim

**Affiliations:** 1 Department of Life Sciences, Pohang University of Science and Technology, Pohang, Republic of Korea; 2 School of Biological Sciences, Nanyang Technological University, Singapore, Singapore; 3 Division of Integrative Biosciences and Biotechnology, Pohang University of Science and Technology, Pohang, Republic of Korea; 4 The Graduate School of Biotechnology and Plant Metabolism Research Center, Kyung-Hee University, Suwon, Republic of Korea; Winship Cancer Institute of Emory University, United States of America

## Abstract

Uncontrolled proliferation, a major feature of cancer cells, is often triggered by the malfunction of cell cycle regulators such as protein kinases. Recently, cell cycle-related protein kinases have become attractive targets for anti-cancer therapy, because they play fundamental roles in cellular proliferation. However, the protein kinase-targeted drugs that have been developed so far do not show impressive clinical results and also display severe side effects; therefore, there is undoubtedly a need to investigate new drugs targeting other protein kinases that are critical in cell cycle progression. Vaccinia-related kinase 1 (VRK1) is a mitotic kinase that functions in cell cycle regulation by phosphorylating cell cycle-related substrates such as barrier-to-autointegration factor (BAF), histone H3, and the cAMP response element (CRE)-binding protein (CREB). In our study, we identified luteolin as the inhibitor of VRK1 by screening a small-molecule natural compound library. Here, we evaluated the efficacy of luteolin as a VRK1-targeted inhibitor for developing an effective anti-cancer strategy. We confirmed that luteolin significantly reduces VRK1-mediated phosphorylation of the cell cycle-related substrates BAF and histone H3, and directly interacts with the catalytic domain of VRK1. In addition, luteolin regulates cell cycle progression by modulating VRK1 activity, leading to the suppression of cancer cell proliferation and the induction of apoptosis. Therefore, our study suggests that luteolin-induced VRK1 inhibition may contribute to establish a novel cell cycle-targeted strategy for anti-cancer therapy.

## Introduction

Tumorigenesis is associated with a dysregulation of cell division, which is often triggered by defects in the regulation of protein modulators that play critical roles in cell cycle checkpoints and progression [1]. Among the proteins that make up the cell cycle machinery, recent therapeutic strategies have attempted to take advantage of targeting several cell cycle protein kinases to enhance drug selectivity and therapeutic effectiveness [2], [3]. Accordingly, such cell cycle-related protein kinases have become attractive targets for anti-cancer therapy, owing to their fundamental functions in controlling cell growth. For instance, small-molecule inhibitors of the DNA damage checkpoint proteins Chk 1 and 2 were used with the intention of causing cell cycle arrest and apoptosis during interphase [4]–[6]. In addition, some mitotic inhibitors targeting the cyclin-dependent kinase (CDK) family, Aurora kinases, and Polo-like kinases have been developed to provoke impeded mitotic entry, mitotic arrest, and mitotic catastrophes by causing deficiencies in chromosome condensation, chromosome alignment, spindle formation, and the spindle assembly checkpoint [1]–[3]. For several promising inhibitors, clinical trials have already been conducted to develop a novel class of anti-cancer drugs. In the clinical trial stages, unfortunately, their clinical efficacy did not show impressive results, but rather elicited limited responses or even unexpected severe side effects [3]. Nevertheless, effective inhibition of certain phase of cell cycle is still regarded as a valuable strategy to treat cancer, Thus identification of novel, cell cycle-specific, druggable target proteins and the development of their selective inhibitors that might have potential to become chemotherapeutic agents are undoubtedly required.

In this regard, we investigated whether Vaccinia-related kinase 1 (VRK1) might be an adequate molecular target in accordance with the cell cycle targeting strategy. VRK1, which specifically phosphorylates serine and threonine residues, is a mitotic kinase that plays an important role in cell cycle progression by participating in wide variety of cell division processes [7], [8]. VRK1 expression is specifically abundant in highly proliferative cells such as fetal and tumor tissues, and mainly displays a tendency to upregulate during the mitotic phase in the cell cycle [9], [10]. In the G1/S phases, VRK1 promotes cyclin D1 (CCND1) expression to induce the G1/S transition by phosphorylating cAMP response element (CRE)-binding protein (CREB) and thereby enhancing the binding affinity of CREB to the CCND1 promoter [11]. Furthermore, VRK1 takes part in nuclear envelope (NE) dynamics such as NE assembly/disassembly via phosphorylation of barrier-to-autointegration factor (BAF) during interphase and mitotic entry/exit [12]. BAF is a chromatin-associated protein functioning as a link between DNA and the NE [13]. The dynamic status of BAF during cell cycle progression is tightly regulated by VRK1 activity; BAF phosphorylation by VRK1 stimulates chromatin release from NE, and recruits NE-associated proteins into core region during telophase [12], [14]. In the mitotic phase, VRK1 affects histone modification by phosphorylating histone H3 [10]. Phosphorylation of histone H3 Ser10 by VRK1 and other several mitotic kinases is a well-known histone code inducing chromatin condensation at mitotic entry or the G2/M phase transition. In the cell cycle, VRK1-mediated histone H3 phosphorylation is influenced by other regulators. Mitogen-activated protein kinase phosphatase 2 (MKP2), a dual-specificity phosphatase that inactivates MAP kinases (MAPKs), plays a role as a negative regulator of VRK1-mediated histone H3 phosphorylation at the mitotic phase [15]. During interphase, Macro histone H2A1.2 (MacroH2A1), a core histone variant, suppresses the approach of VRK1 to histone H3 by sequestration [16].

Additionally, recent studies also have shown the significance of VRK1 in the process of cell proliferation. VRK1 has a critical role not only in somatic cell proliferation, but also in germ cell development [17], [18]. Moreover, VRK1 also plays a role in the maintenance of the telomere by phosphorylating hnRNP A1, which stimulates telomerase and binds to the telomere DNA sequence [19]. VRK1 is required for G0 exit, Golgi fragmentation, DNA damage-induced apoptosis, and stress responses, which are necessary to maintain cell cycle progression [20]–[24]. Thus, considering the cell cycle-related characteristics of VRK1, the identification of VRK1 inhibitor is required for anti-cancer therapies. Although it is reported that some drugs to inhibit other protein kinases might inhibit the kinase activity of VRK1 [25], the inhibitory mechanism and anti-cancer effects through VRK1 inhibition were still elusive.

In our study, we screened a small-molecule natural compound library and identified luteolin (3′,4′,5,7-tetrahydroxyflavone) as the small-molecule to inhibit VRK1 kinase activity. Luteolin is a naturally occurring flavonoid that is commonly distributed in plants and is well-known to act as a potent antioxidant [26]. In traditional Asian remedies, luteolin-abundant herbs have been used as traditional medicines for treating many disorders such as painful conditions, inflammatory diseases, hypertension, and cancer [27]. Today, many lines of evidence have demonstrated numerous pharmacological effects of luteolin including anti-cancer, anti-inflammation, and anti-allergy activities [27], [28]. Although it has been ascertained that the anti-cancer properties of luteolin are associated with pro-apoptotic effects, anti-proliferative effects, and inhibition of angiogenesis and metastasis, the molecular mechanisms underlying its anti-cancer activities have not been fully determined [29], [30].

Here, we confirmed that luteolin displays significantly reduced phosphorylation of the cell cycle-related substrates histone H3 and BAF, and directly interacts with VRK1, specifically docking at its catalytic domain. Moreover, we demonstrated that luteolin could induce cell cycle arrest by inhibiting VRK1 kinase activity, leading to the suppression of cancer cell growth and apoptosis. Therefore, we suggest that luteolin is an inhibitor of VRK1, which is one of good candidates to treat cancer.

## Materials and Methods

### Chemicals and Reagents

Luteolin was of analytical grade and purchased from Sigma-Aldrich (St. Louis, MO, USA). Eupatilin and Wogonin were provided from Dr. Baek's Lab in Kyung-Hee University. These compounds were prepared in DMSO (Sigma-Aldrich). [^32^P-γ] ATP was purchased from Perkin Elmer/NEN (Waltham, MA, USA) and recombinant histone H3 was purchased from Roche Applied Science (Indianapolis, IN, USA). Other recombinant proteins such as glutathione sulfotransferase (GST), GST-VRK1, GST-aurora kinase B (AURKB) and His-BAF were expressed in *E.coli* (BL21) and were purified by affinity chromatography. Lamin B, glyceraldehyde 3-phosphate dehydrogenase (GAPDH) and GST antibody was purchased from Santa Cruz Technology (Santa Cruz, CA, USA) and histone H3 phospho-Ser10 antibody was purchased from Abcam (Cambridge, UK) and those against histone H3, phospho-CREB and CREB were obtained from Cell Signaling Technology (Danvers, MA, USA). VRK1 and phospho-BAF (gift from Robert Craigie, NIH, USA) antibody were generated in rabbit using purified VRK1 proteins. Hoechst 33342 was purchased from Sigma-Aldrich. CNBr-Sepharose 4B was purchased from Sigma-Aldrich.

### Cell culture and transfection

BEAS-2B cells were obtained from the ATCC (Manassas, VA). Previously described HeLa [31], HEK293A [16], SH-SY5Y [32], U2OS [32], and A549 [31] cells were used in this study. The human cervical adenocarcinoma cell line HeLa, human embryonic kidney cell line HEK293A, the human bronchial epithelial cell line BEAS-2B and the human neuroblastoma cell line SH-SY5Y were grown in Dulbecco's modified Eagle medium (DMEM) medium supplemented with 10% fetal bovine serum and 1% penicillin-streptomycin. The human osteosarcoma cell line U2OS and the human lung adenocarcinoma cell line A549 were grown in RPMI1640 medium supplemented with 10% fetal bovine serum and 1% penicillin-streptomycin. These cell lines were maintained at 37°C in a humidified atmosphere of 5% CO2. Transient transfection of HeLa and U2OS cells was carried out using an MP-100 microporator (Invitrogen, Carlsbad, CA, USA) according to the manufacturer's protocol.

### Protein kinase assay

An in vitro kinase assay was performed in accordance with the methods previously described [31]. In brief, an in vitro kinase assay was carried out in the kinase buffer containing GST-VRK1, GST-AURKB, His-BAF, histone H3, and [32P-γ] ATP. The kinase assay mixtures were incubated at 30°C for 30 min and radioactive incorporation was detected by autoradiography. The quantities of proteins used in kinase assays were measured by using silver nitrate (Sigma-Aldrich) or coomassie blue (Sigma-Aldrich).

### Cell viability assay

Cells were treated for 24 h or 48 h with flavonoids (luteolin, eupatilin, and wogonin) or with dimethylsulfoxide (DMSO) as a control. Cell viability was assessed using the 3-(4,5-dimethylthiazol-2-yl)-2,5-diphenyltetarazolium bromide (MTT) assay according to the manufacturer's protocol. The half-maximal inhibitory concentration (IC50) was determined using Graphpad Prism (GraphPad, San Diego, CA, USA).

### Luteolin-Sepharose 4B preparation and in vitro pull-down assay

Sepharose 4B bead powder was suspended in activation solution for coupling reaction. Activated Sepharose 4B beads were incubated in coupling solution with luteolin on a rotary shaker at 4°C overnight. For the in vitro pull down assay, GST-VRK1 and GST were incubated with luteolin-Sepharose 4B beads in reaction solution for 12 h. Proteins bound to the beads were analyzed by immunoblotting. We carried out the detailed procedures which were described previously [33].

### Surface Plasmon Resonance (SPR) assay

The kinetic parameters of the interaction between VRK1 and luteolin were evaluated by an SPR assay using the automatic SR7500DC system (Reichert Technologies, Depew, NY, USA). For preparation of the VRK1-conjugated gold chip, VRK1 was immobilized on a CMDH chip (#13206066) and then luteolin, eupatilin and wogonin were injected on the surface of chip at the indicated concentrations, diluted in 10 mM phosphate-buffered saline (PBS) containing 1% DMSO as a running buffer. Analysis of the collected data was performed by scrubber2 software (BioNavis, Tampere, Pirkanmaa, Finland).

### Ligand docking assay

A homology model developed from the X-ray structure of VRK1 was employed to assess the molecular interaction and the binding mode of newly identified VRK1 leads. In the present study, the model structure was energy-minimized for 5,000 steps by the CHARM force-field and conjugate gradient method in the Discovery Studio 3.0 suite [34]. The 3-D coordinates of luteolin were prepared using the Prepare Ligand module and energy-minimized for 2,000 steps using the Smart Minimizer algorithm in the Discovery Studio 3.0 suite. The molecular docking program GOLD 5.0 was employed to assess the binding mode of the VRK1 leads. Our previous NMR binding studies have identified the key residues that are perturbed upon ligand binding; these were used to define the active site [34]. Default settings and scoring functions, the GOLD PLP and GOLD scoring function, were employed to score docking interactions and their probable docking mode of binding.

### Flow cytometry assay

For cell cycle analysis, HeLa cells were transfected with GFP, GFP-VRK1, GFP-BAF and then treated with DMSO (vehicle) and 10 µM luteolin. Afterward, cells were fixed with 70% ethanol containing 0.4% Tween-20 and stained with propidium iodide in PBS. The cell cycle and cellular DNA contents were analyzed by flow cytometry in a FACSCalibur flow cytometer (BD Biosciences, Franklin Lakes, NJ, USA). Data acquisition was performed with the Cell Quest program (BD Biosciences). For apoptosis analysis, HeLa cells were treated with luteolin in a concentration-dependent manner and double stained with propodium iodide and Annexin-V allophycocyanin. Cells were analyzed by flow cytometry.

### Immunofluorescence staining

HeLa cells were transfected with red fluorescent protein (RFP), RFP-VRK1, GFP, and GFP-BAF using microporation, and then treated with 10 µM of luteolin for 24 h. Subsequently, cells were fixed in 4% paraformaldehyde for 20 min and blocked with 10% FBS in PBS for 1 h at room temperature. Cells were stained with Hoechst 33342 in PBS for 20 min at room temperature and viewed using a confocal laser-scanning microscopy (Fluoview FV1000; Olympus, Tokyo, Japan). HeLa cells were treated with or without luteolin(10 µM) for 24 hr. Subsequently, we carried out detailed procedures which were described, and then the cells were stained with lamin B antibody and viewed using Zeiss fluorescence microscope (Carl Zeiss Ltd., Jena, Germany) or confocal laser-scanning microscopy.

### Quantitative reverse transcription (RT)-PCR

Total RNA from HeLa cells was prepared using the TRI agent (Molecular Research Center, Cincinnati, OH, USA) according to the manufacturer's instructions and then reverse transcribed to generate complementary DNA. Quantitative RT-PCR was performed using the SYBR Green PCR mixture (Takara Bio Inc., Shiga, Japan) and a real-time detector system (Applied BioSystems, Foster City, CA USA). GAPDH level was used to normalize transcript levels.

## Results

### Luteolin is a potent inhibitor of VRK1 kinase activity

**Figure 1:**
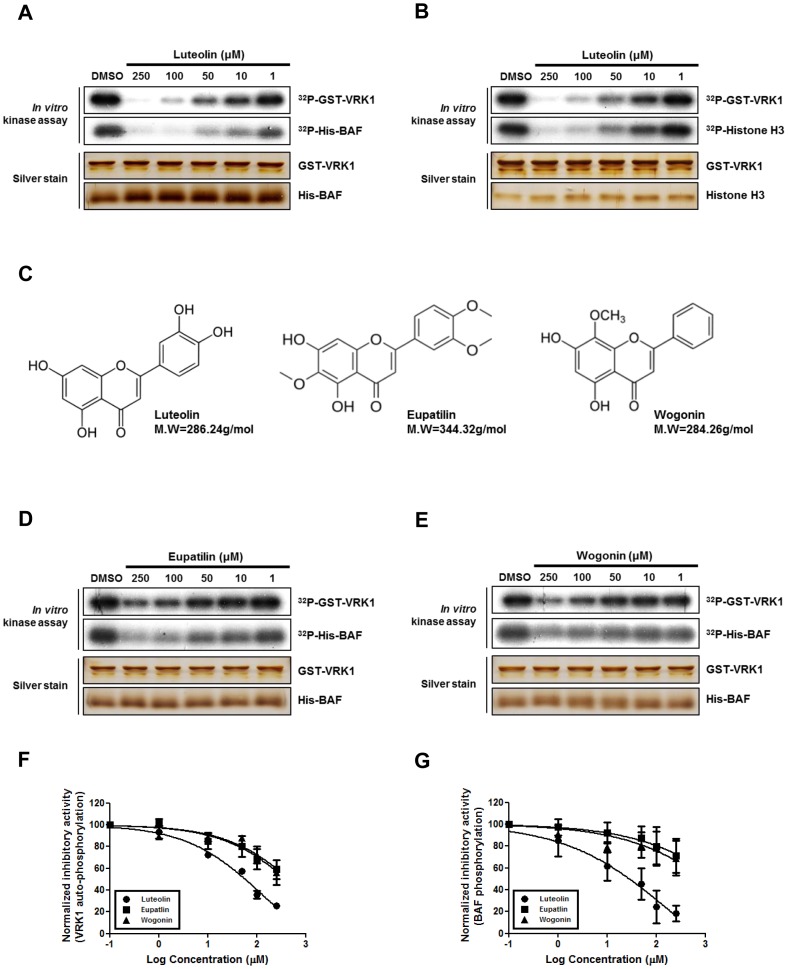
Inhibitory effects of luteolin on VRK1 kinase activity. (A) and (B), *in vitro* kinase activity measurements for VRK1 with BAF (A) or VRK1 with histone H3 (B) were performed by increasing concentrations of luteolin (0.0, 1.0, 10, 50, 100, and 250 µM), and then VRK1 and substrate proteins were stained with silver nitrate. (C), Chemical structures and molecular weights of luteolin, eupatilin, and wogonin. (D) and (E), *in vitro* kinase assay for VRK1 with BAF were performed by increasing concentrations (0.0, 1.0, 10, 50, 100, and 250 µM) of eupatilin (D) or wogonin (E), and then VRK1 and BAF proteins were stained with silver nitrate. (F) and (G), Quantification of VRK1 auto-phosphorylation (F) or BAF phosphorylation (G) described in (B), (D) and (E). Data in (F) and (G) represent means of three independent experiments ±SEMs.

To examine whether luteolin could effectively suppress the enzymatic activity of VRK1, we performed an *in vitro* kinase assay and compared the inhibitory effects of luteolin with those of other flavonoid-like compounds. Luteolin significantly inhibited the VRK1-mediated phosphorylation of BAF and histone H3 in a dose-dependent manner. Moreover, the auto-phosphorylation activity of VRK1 was attenuated by treatment with luteolin in a dose-dependent manner (Fig. 1A and B). Although eupatilin and wogonin, flavonoid-like chemical compounds that show structural similarity to luteolin (Fig. 1C), weakly inhibit VRK1 kinase activity (Fig. 1D and E), luteolin showed the most prominent inhibitory effects on BAF phosphorylation and VRK1 auto-phosphorylation, suggesting the specificity of luteolin. The specificity of luteolin for VRK1 was further confirmed by its normalized inhibitory effects on BAF phosphorylation and VRK1 auto-phosphorylation (Fig. 1F and G). These results indicate that luteolin is a potent inhibitor of VRK1 kinase activity, which is required for the modulation of cell cycle progression.

### Luteolin directly interacts with VRK1

**Figure 2:**
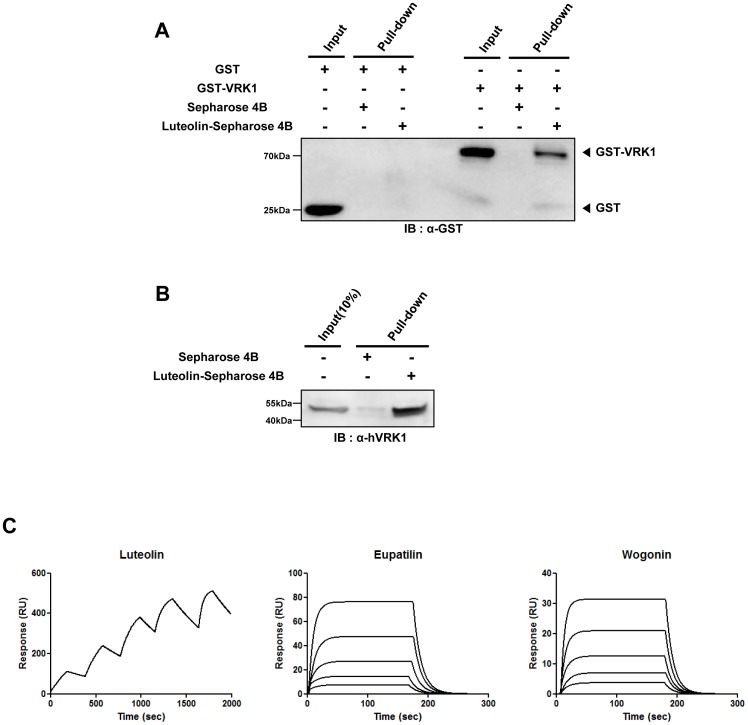
Direct interaction of luteolin with VRK1. (A), Pull-down assay using luteolin-conjugated sepharose 4B beads or control sepharose 4B beads with recombinant VRK1 protein. Purified GST and GST-VRK1 proteins are incubated with indicated beads, and then pull down assay was performed. Each proteins were detected by immunoblotting with GST antibody. (B), Pull-down assay using luteolin conjugated sepharose 4B beads with SH-SY5Y cell lysate. Cell lysate are incubated with indicated beads, and then pull-down was performed. The proteins were detected by immunoblotting with VRK1 antibody. (C), SPR detection for the interaction of luteolin with VRK1. The data were obtained by kinetic titration method with sequentially injection of analytes without regeneration steps. Data for eupatilin and wogonin were obtained by classical methods.

Because luteolin suppressed the action of VRK1 towards its substrates, we hypothesized that luteolin might inhibit kinase activity through direct binding to VRK1. To investigate the interaction between luteolin and VRK1, we conducted a pull-down assay using luteolin-conjugated sepharose beads. GST could not bind to either luteolin-conjugated sepharose beads or control beads. However, GST-VRK1 successfully bound to luteolin-conjugated sepharose beads, but did not bind to control beads that were not conjugated to luteolin (Fig. 2A). We next performed a pull-down assay with SH-SY5Y cell lysates to inspect whether luteolin binds to endogenous VRK1 in addition to recombinant VRK1 (Fig. 2B). Endogenous VRK1 could also bind to luteolin-conjugated sepharose beads. These results suggest that luteolin directly interacts with VRK1 *in vitro*.

Direct interaction between luteolin and VRK1 was further analyzed using surface plasmon resonance (SPR) to monitor the binding kinetics. Recombinant VRK1 proteins were covalently immobilized on a sensor chip as a ligand; subsequently, luteolin, eupatilin and wogonin were added in a concentration-dependent manner as an analyte (Fig. 2C). Kinetic parameters of the interaction between the VRK1-coated sensor chip and these analytes were evaluated by software and are represented in Table 1. Calculated constants of these analytes towards VRK1 exhibit that luteolin has most strong binding affinity among them. Taken together, these results indicate that luteolin directly binds to VRK1 with high binding affinity.

**Table 1:** Kinetic parameters of the binding of luteolin to VRK1.

	K_a_(M^−1^S^−1^)	K_d_(S^−1^)	K_D_
Luteolin	43.62	0.0464	5.8 µM
Eupatiln	414	0.0763	184 µM
Wogonin	51.72	0.083	120 µM

### The inhibitory effect of luteolin is mediated by its direct binding to the catalytic domain of VRK1

**Figure 3:**
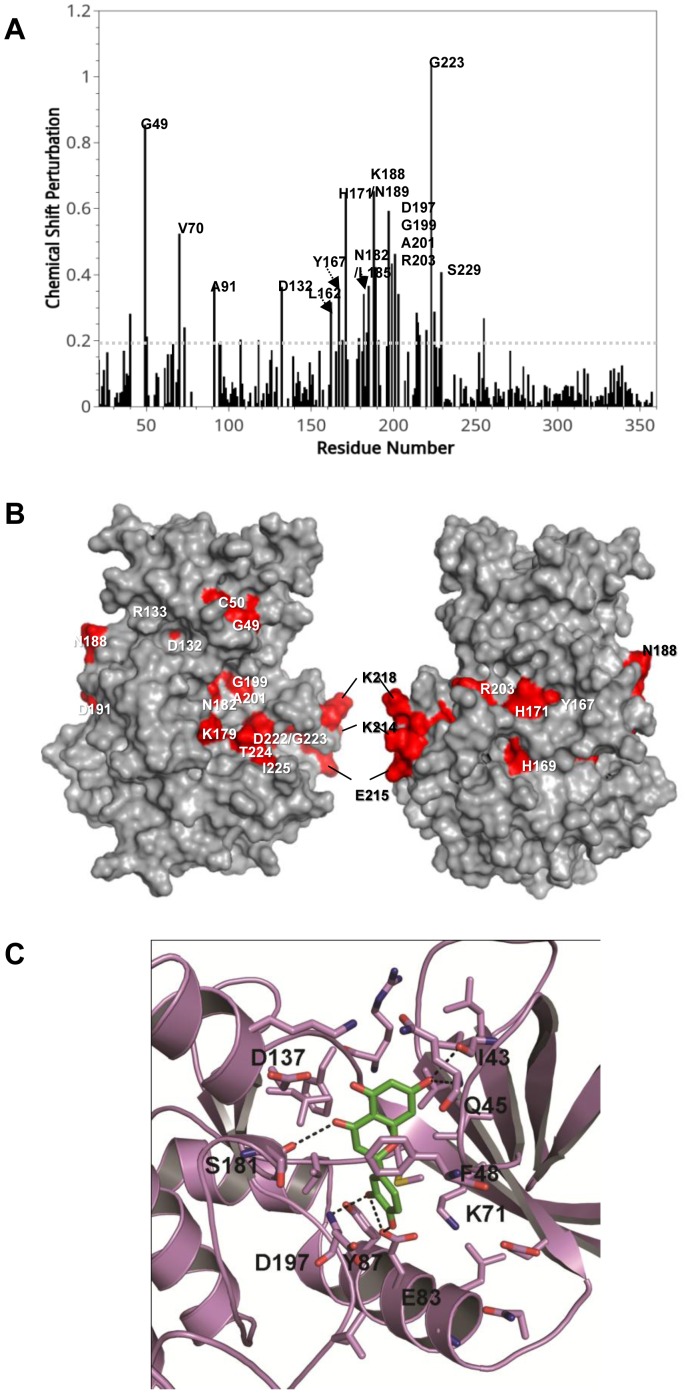
NMR titration assay and in silico modeling for interaction of luteolin with VRK1. (A), NMR titration experiments were performed. Spectrum of chemical shift perturbations versus amino acid residues of the VRK1 protein after binding of luteolin. (B), Mapping of chemical shift perturbations on the VRK1 protein. Most of the perturbed residues (shown in red) are located close to the catalytic domain of VRK1. (C), *in silico* modeling of the binding mode of luteolin to the VRK1 protein. Luteolin is predicted to fit in the vicinity of the G-loop, catalytic site, and α-C lobe.

To determine the binding region of VRK1 for luteolin, the interaction between luteolin and VRK1 was assessed by NMR titration experiments and *in silico* modeling. Amino acid residues affected by interaction with luteolin showed dramatically increased chemical shift perturbations (Fig. 3A). These residues are represented in the spectrum of chemical shift perturbations and also assigned on the molecular map of VRK1 (Fig 3B), suggesting that these residues are mainly located in the vicinity of catalytic domain, which is involved in ATP binding [34].

In *in silico* modeling of the binding mode of luteolin toward VRK1, luteolin is predicted to fit in the vicinity of the G-loop, catalytic site, and α-C lobe (Fig. 3C). The 2, 3-dihydroxyl moieties of the 2-phenyl group on the chromene scaffold interact with the E83 residue of α-C lobe. Further, the 2-hydroxyl group also interacts with the D197 residue of the catalytic loop. Similarly, the 4-keto group on the chromene scaffold interacts with S181 via a hydrogen-bonding interaction. The 7-hydroxyl moiety on the chromene scaffold also makes hydrogen-bonding interactions with the I43 and Q45 residues from the G-loop. Apart from these hydrogen-bonding interactions, the chromene scaffold also has strong stacking hydrophobic interactions with the F48 residue of the G-loop and the hydrophobic residues surrounding the active site (not shown). Luteolin has most of its key interactions with the G-loop and catalytic site residues, which firmly stabilize the luteolin molecule into the active site and inhibit its kinase activity.

### Luteolin induces cell cycle arrest via the inhibition of BAF phosphorylation by VRK1

**Figure 4:**
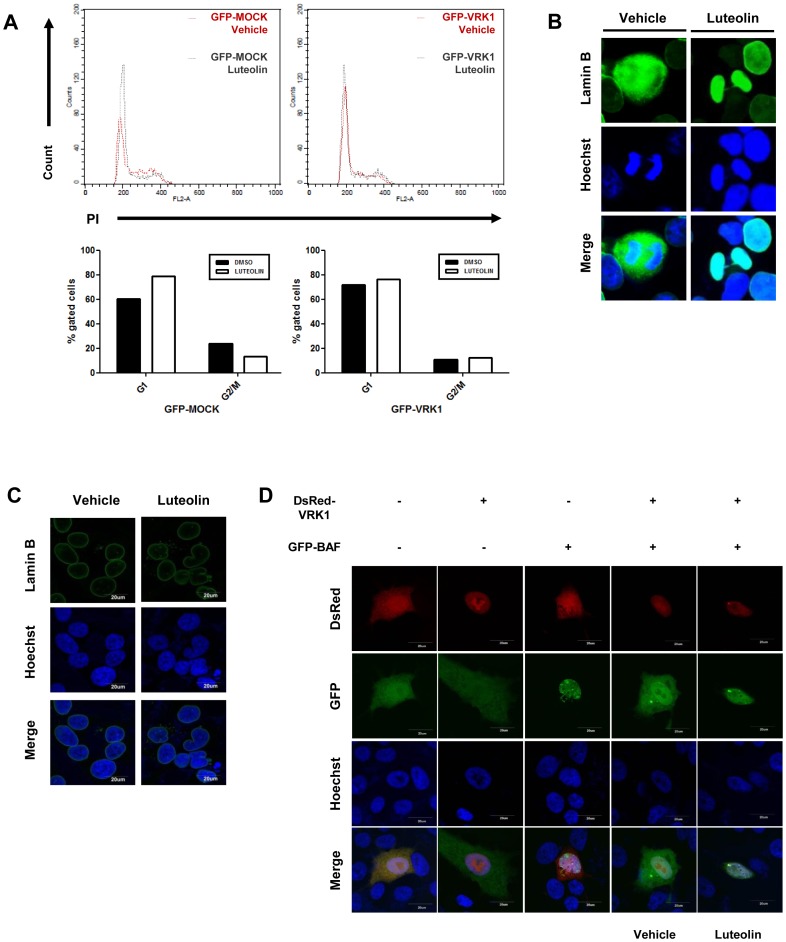
Luteolin-induced cell cycle arrest and nuclear envelope disassembly defects. (A), Cell cycle analysis was carried out by flow cytometry. HeLa cells transfected with GFP or GFP-VRK1 were treated with luteolin for 24 hours, and 10,000 cells were gated for analysis. Quantitative data are below the histogram plots. (B) and (C), HeLa cells treated with vehicle or luteolin were stained with lamin B antibody to visualize nuclear envelope and with Hoechst 33342 to visualized DNA. Alexa 488 dye-conjugated antibody was used as secondary antibody. The slides were visualized by fluorescence microscopy (B), or confocal laser scanning microscopy (C). (D), Fluorescent staining of VRK1, BAF, and DNA for analysis of the phosphorylation-mediated re-localization of BAF. Cells co-transfected with GFP or GFP-BAF with RFP or RFP-VRK1 were treated with DMSO or 10 µM luteolin for 24 hours.

Luteolin has been demonstrated previously to induce cell cycle arrest at the G1/S and G2/M phase transitions [35]–[39]. However, the molecular mechanism of cell cycle arrest by luteolin is unknown, except for the possibility that Aurora B kinase might be a molecular target of luteolin to regulate cell cycle progression, based on evidence that Aurora B is inhibited by luteolin and plays a role as a key mediator of mitotic progression [40]. VRK1 is another mitotic kinase that has been known to carry out coordinating roles in cell cycle progression by the phosphorylation of several cell cycle-related substrates such as BAF, histone H3, and CREB [10]–[12]. Thus, we evaluated whether luteolin induces cell cycle arrest by inhibiting VRK1. PI-stained cells treated with DMSO or luteolin were analyzed by flow cytometry to assess the cell cycle. Upon treatment with luteolin, the population in the G1 phase was significantly increased compared to that in DMSO-treated cells (Fig. 4A, left panel). In contrast, the increased G1-phase population disappeared upon overexpression of GFP-tagged VRK1 (Fig 4A, right panel), indicating that luteolin causes arrest in the early stages of cell cycle by the inhibition of VRK1.

As mentioned above, VRK1 participates in the phosphorylation-mediated re-localization of BAF to the cytoplasm, chromatin condensation via histone H3 phosphorylation, and CCND1 expression by CREB phosphorylation to facilitate cell cycle progression [10]–[12]. To further explain the mechanism of cell cycle arrest by luteolin-induced interference with VRK1 activity, we evaluated whether luteolin disturbs these processes. Because it has been demonstrated that VRK1 enhances the expression of CCND1, which is correlated with G1/S progression, through increased activity of the CRE element in the CCND1 promoter by phosphorylated CREB binding, we speculated that CREB phosphorylation and the mRNA level of CCND1 may be affected by luteolin treatment. However, there were no significant differences in CREB phosphorylation and the mRNA level of CCND1 between DMSO and luteolin treatment (Fig. S1), implying that luteolin-induced G1/S arrest may have been a consequence of defects in other processes rather than suppression of CREB phosphorylation.

We next tested the possible involvement of BAF in G1/S arrest caused by luteolin. In *Drosophila* and *C. elegans*, it is well established that BAF has fundamental roles in the organization of nuclear structure and cell cycle progression [13], [41]. In addition, the nuclear localization of BAF influences cell cycle progression in human cells; VRK1, the only identified upstream kinase of BAF, can alter BAF localization by phosphorylation to induce the release of BAF from both DNA and LEM-domain proteins such as Lap2, emerin, and MAN1 [12], [14], [31], [42]. VRK1-mediated BAF re-localization is essential process for DNA release during G1/S transition. To detect any perturbations caused by luteolin in nuclear envelope dynamics in the cell cycle progression, we stained the nuclear envelope with the nuclear lamin B antibody. Lamin B is detached from chromatin in mitosis, however, loss of VRK1 or expression of BAF mutant perturbs chromatin separation from NE [12], [42], [43]. We observed whether nuclear lamin B was not dispersed by treatment with luteolin. Nuclear lamin B was released into the cytoplasm at late anaphase as shown in vehicle-treated cells, while lamin B is stuck into chromosomes at same phase as shown in luteolin-treated cells (Fig. 4B), suggesting that luteolin-induced VRK1 inhibition disturbs VRK1-mediated BAF phosphorylation.

In addition, we further found that luteolin caused abnormal nuclear envelope morphology such as invagination and bleb (Fig. 4C), which is a phenomenon to be induced by depletion of VRK1 [44]. Thus, we suggest that luteolin-mediated attenuation of BAF phosphorylation might give rise to VRK1 depletion-induced abnormal nuclear morphologies. To confirm that luteolin disturbs VRK1-mediated BAF phosphorylation, we observed phospho-BAF level using immunoblotting. Phospho-BAF level was decreased in 10 µM luteolin-treated cell lysate (Fig. S2), indicating that VRK1 catalytic activity is reduced by luteolin *in vivo*.

A previous study showed that ectopic expression of VRK1 results in BAF re-localization to the cytoplasm [12], [31]. Thus, we further investigated whether the phenomenon of BAF re-localization was affected by luteolin-mediated inhibition of VRK1. When both RFP-VRK1 and GFP-BAF were introduced together, BAF appeared to be dispersed throughout the cell, as has been reported previously [31]. However, we confirmed that this phenomenon was disrupted by treatment with luteolin (Fig. 4D). The localization of GFP-BAF was restricted to the nucleoplasm in spite of VRK1 overexpression, suggesting that luteolin treatment effectively reduced BAF phosphorylation by inhibiting VRK1, followed by cell cycle arrest. To examine the role of BAF during cell cycle, we analyzed DNA contents after luteolin treatment. The ectopic expressed BAF does not influence cell cycle progression (Fig. S3A). Upon luteolin administration, ectopic expressed BAF could not rescue luteolin-induced G1 accumulation (Fig. S3B), suggesting that luteolin-induced G1 arrest is not resulted by BAF inhibition but it by inhibition of VRK1-mediated BAF phosphorylation.

### Luteolin induces reduced cell viability and subsequent apoptosis

**Figure 5:**
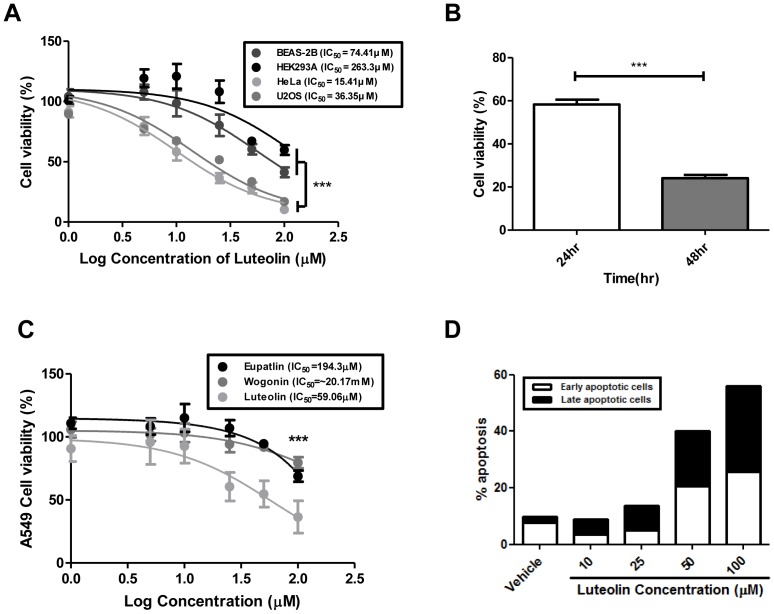
Luteolin exhibits selective cytotoxicity towards tumorigenic cells and induces apoptosis. (A), The effects of luteolin on cell viability of tumorigenic (HeLa and U2OS) and non-tumorigenic (BEAS-2B and HEK293A) cell lines. Cells were treated with indicated concentration of luteolin for 24 hours. Afterward, cell viability was analyzed by the MTT assay. (B), Time-dependent effects of luteolin on HeLa cell viability. HeLa cells were treated with indicated luteolin 10 µM for indicated times. Cell viability was assessed by the MTT assay. Error bars represents means ±SEM (n = 10). Symbols (***) represent p value <0.0001. (C), The effects of flavonoids on A549 cell viability were compared. Error bars represent means ±SEM (n = 10). Symbols (***) represent p value <0.0001. The P-values in (A), (B) and (C) were calculated using Student's t-test. (D), Proapoptotic effect of luteolin on HeLa cells. HeLa cells treated with indicated concentrations of luteolin were double stained with PI/Annexin-V allophycocyanin. Apoptosis analysis was performed by flow cytometry.

Once the inhibitory effect of luteolin mediated by binding to the catalytic domain of VRK1 was confirmed, we evaluated its anti-tumor effects on tumor cell proliferation and survival. The effects of luteolin on cell viability were measured by the MTT assay at different concentrations. We observed that luteolin treatment significantly reduced the growth of the tumorigenic cell lines HeLa and U2OS compared to the non-tumorigenic cell lines BEAS-2B and HEK293A (Fig. 5A). The IC_50_ values in HeLa and U2OS cells were determined as 15.41 µM and 36.35 µM, respectively, whereas those in BEAS-2B and HEK293A cells were increased several-fold compared to tumorigenic cells (74.41 µM and 263.3 µM, respectively). This result shows that the inhibitory effect of luteolin on cell proliferation is more pronounced in tumorigenic cells than non-tumorigenic cells, although VRK1 protein level in cell lines is not correlated with tumorigenicity (Fig. S4). Furthermore, luteolin inhibits the cell viability of HeLa cells in a time-dependent manner (Fig. 5B).

VRK1 has been focused on a suitable target for lung cancer treatment because VRK1 is related to cell cycle and DNA damage response in lung cancer tissue, not normal tissue [45]. Thus, we measured the cytotoxicity effects of luteolin in A549, lung cancer cell line. The IC_50_ in A549 is similar to these in HeLa and U2OS cell, and shows a more pronounced effect on tumor cell growth than eupatilin and wogonin (Fig. 5C). These indicate that among flavonoid-like compounds, luteolin has the most specific effect on cancerous cells.

We further investigated whether the reduced cell proliferation upon luteolin treatment accompanied the induction of apoptosis. PI/Annexin-V allophycocyanin double staining was used to analyze the pro-apoptotic effect of luteolin. Upon luteolin administration, the numbers of early and late apoptotic cells are increased in a concentration-dependent manner (Fig. 5D).

## Discussion

Unlimited proliferation is a major hallmark of tumor cells and is closely related to an uncontrolled cell cycle. Multiple lines of evidence suggest that there is an intimate connection between tumor development and cell cycle dysregulation [46]. So far, the CDK family and other mitotic kinases have been considered as potent pharmacological targets for anti-cancer therapy [1]–[3]. However, because they play pivotal roles in not only tumorigenic cells but also normal cells, and because cell cycle progression is also a critical process in normal cells, these kinases are unsuitable for drug targets from clinical trials [3]. On the other hand, VRK1 is a mitotic kinase that is expressed in normal cells like other mitotic kinases, but it has significant function in the growth of some tumors such as lung cancer and head and neck squamous cancer [8], [45]. In studies of the rewiring of the mitotic network, VRK1 was identified as a critical mitotic protein kinase in proliferation of lung cancer. It means that VRK1 is a potential druggable target in the lung cancer-specific mitotic network, because its gene expression is specific for the cancer cell cycle network and correlates with cell cycle markers [45]. Moreover, when VRK1 is downregulated, it causes G1 cell cycle arrest and reduced proliferation of tumor cells. These results are similar to our observations in this study (Fig. 4 and 5). There are several lines of evidence supporting an important role of VRK1 in tumor biology. First, it is reported that VRK1 gene expression could be selected as a significant prognostic indicator in the development of estrogen receptor-positive breast cancers [47]. In addition, suppressing VRK1-mediated BAF phosphorylation by the small-molecule inhibitor obtusilactone B induces abnormal nuclear envelope dynamics and tumor cell death, consistent with our finding that luteolin-mediated VRK1 inhibition causes similar phenomena [31] (Fig. 4). Another early study suggesting that VRK1 is an upstream kinase for 53BP1 foci formation in response to DNA damage implies that specific VRK1 inhibitors might contribute to additional DNA damage accumulation in tumor cells, thereby leading to tumor-specific cell death [23]. In addition, several studies have demonstrated that VRK1 depletion is able to impair tumor proliferation and metastasis [48]. However, the molecular mechanism of VRK1 inhibition-induced anti-proliferative effects on tumor cells remains to be further described in detail to develop a novel chemotherapy strategy.

In recent studies, natural product-derived compounds have been evaluated as safe and effective chemical substances for treating many disorders. In particular, it has been demonstrated that polyphenolic compounds including luteolin, wogonin, eupatilin, and myricetin have multiple anti-cancer properties such as anti-proliferative, anti-metastatic, anti-angiogenesis, and pro-apoptotic effects [49]–[51]. Although several early studies have found that luteolin attenuates cell cycle progression or induces cell cycle arrest in tumor cells, little is known about the detailed molecular mechanism in cell cycle dysregulation by luteolin [35]–[40]. Interestingly, in our study, we identified VRK1, a core cell cycle regulator in proliferating cells, as a direct molecular targeting partner of luteolin, and further observed the inhibition of cell proliferation and cell cycle perturbations caused by luteolin through suppression of VRK1 activity. Moreover, luteolin showed more selective interaction with VRK1 compared to other flavonoid-like compounds eupatilin and wogonin (Fig. 1 and 2). One possible explanation is that small differences in functional groups might confer specificity and preference towards the VRK1 catalytic domain even though these compounds have same flavonoid backbone structure. In conclusion, our study suggests that luteolin is a small molecule inhibitor of the mitotic kinase VRK1, and induces cell cycle arrest and preferential cell death in tumor cells. Hence, we provide evidence supporting the notion that molecular targeting of VRK1 may contribute to the development of a strategy for alternative anti-cancer chemotherapy.

## Supporting Information

Figure S1 **Luteolin does not inhibit VRK1-mediated CREB phosphorylation and CCND1 expression.** (A) The alterations of phospho-CREB levels and VRK1 levels after luteolin treatment at indicated concentration were determined by immunoblotting with indicated antibodies. Lamin B is used for loading control. (B) The alteration of relative mRNA level of CCND1 after luteolin treatment at indicated concentration was determined by quantitative real-time PCR. mRNA level of CCND1 is normalized by GAPDH mRNA. Error bars indicated the SEM.
(TIF)

Figure S2 **Luteolin disturbs VRK1-mediated BAF phosphorylation** ***in vivo*** **.** HeLa cells were treated with increasing concentration of luteolin (0.0, 1.0, 2.0, 5.0 µM) for 24 hr. Each protein levels were detected by indicated antibodies. GAPDH was used as loading control.
(TIF)

Figure S3 **Ectopic expressed BAF does not influence cell cycle distribution, and does not rescue luteolin-induced G1 phase arrest.** (A) HeLa cells were transfected with GFP or GFP-BAF, and then were stained with PI for analyzing DNA contents. Cell cycle analysis was carried out by flow cytometry. (B) GFP or GFP-BAF overexpressing cells were treated with or without 10 µM luteolin for 24 hours, and then were stained with PI. Cell cycle analysis was performed by flow cytometry.
(TIF)

Figure S4 **Endogenous expression levels of VRK1 and phosphorylation of BAF and Histone H3 in various cell lines (HeLa, U2OS, SH-SY5Y, BEAS-2B, HEK293A, A549).** Each proteins and its phosphorylation level were detected by immunoblotting with indicated antibodies when loaded with 20 µg of each cell extracts.
(TIF)
