# Supplementary figures and images for: Luteolin Suppresses Cancer Cell Proliferation by Targeting Vaccinia-Related Kinase 1
Source: PLoS One. 2014 Oct 13;9(10):e109655. doi: 10.1371/journal.pone.0109655 (PMC4195671; doi:10.1371/journal.pone.0109655)

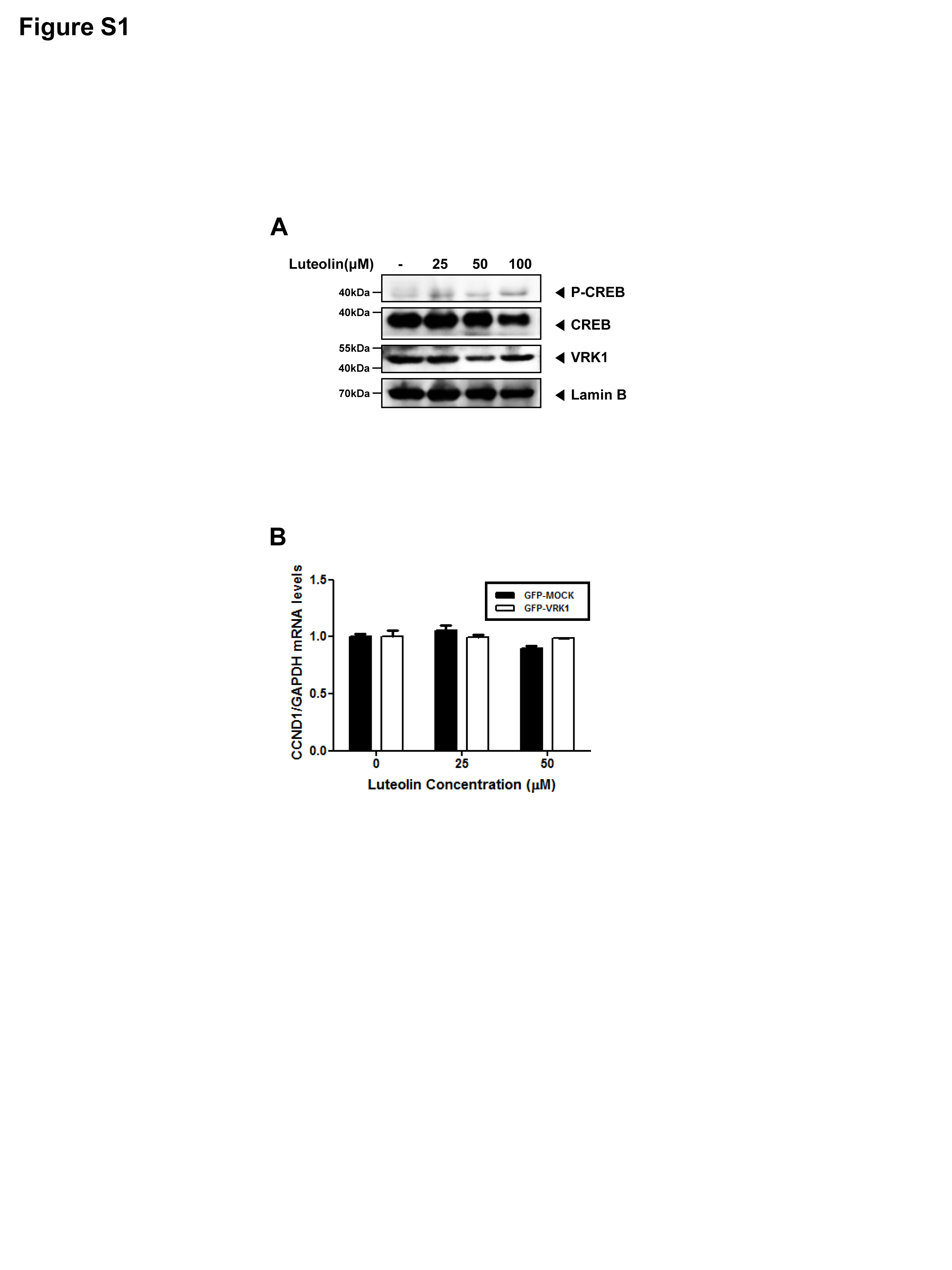

Supplement: Figure S1 — Luteolin does not inhibit VRK1-mediated CREB phosphorylation and CCND1 expression. (A) The alterations of phospho-CREB levels and VRK1 levels after luteolin treatment at indicated concentration were determined by immunoblotting with indicated antibodies. Lamin B is used for loading control. (B) The alteration of relative mRNA level of CCND1 after luteolin treatment at indicated concentration was determined by quantitative real-time PCR. mRNA level of CCND1 is normalized by GAPDH mRNA. Error bars indicated the SEM. (TIF) [file pone.0109655.s001.tif]

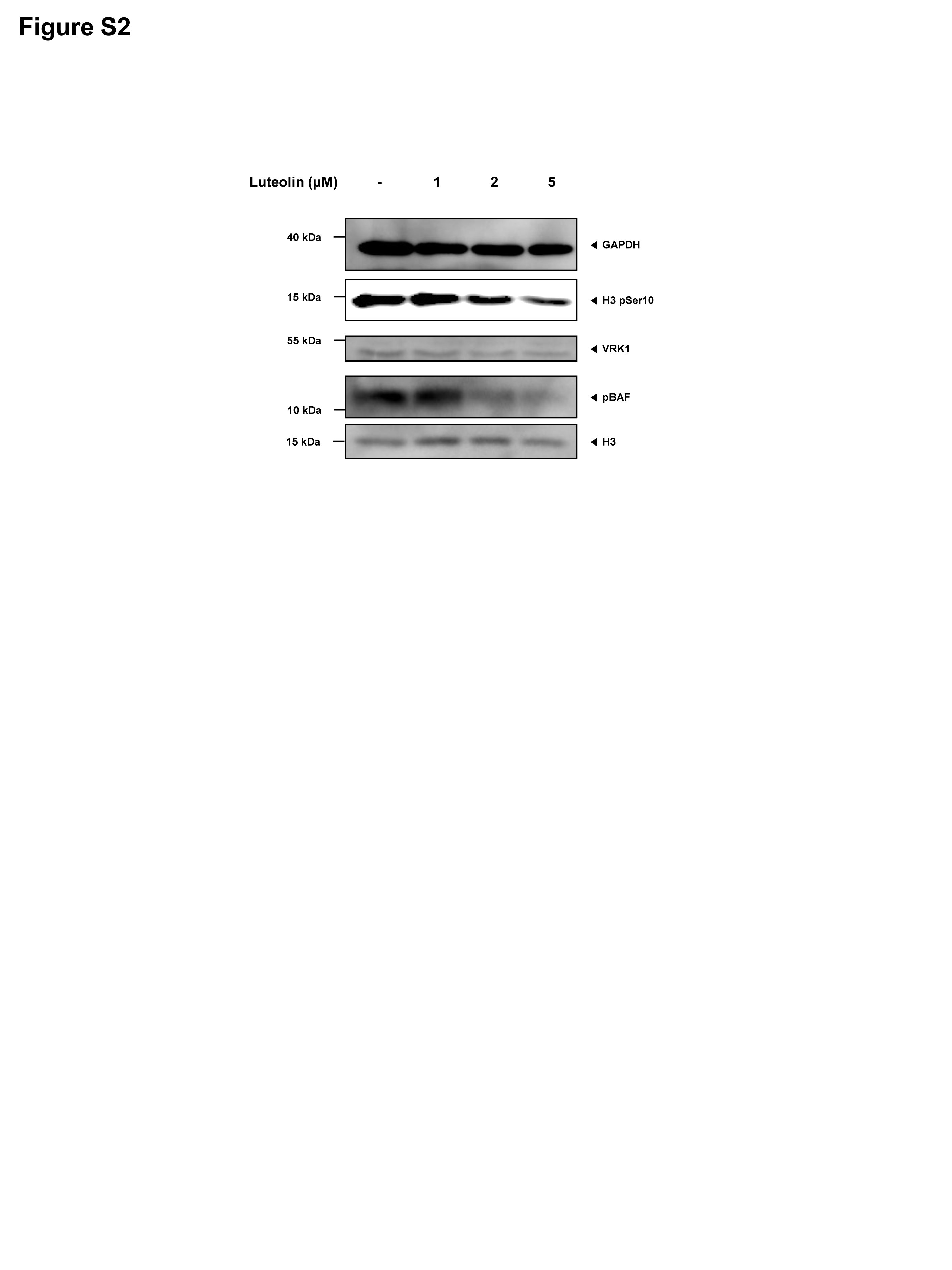

Supplement: Figure S2 — Luteolin disturbs VRK1-mediated BAF phosphorylation in vivo . HeLa cells were treated with increasing concentration of luteolin (0.0, 1.0, 2.0, 5.0 µM) for 24 hr. Each protein levels were detected by indicated antibodies. GAPDH was used as loading control. (TIF) [file pone.0109655.s002.tif]

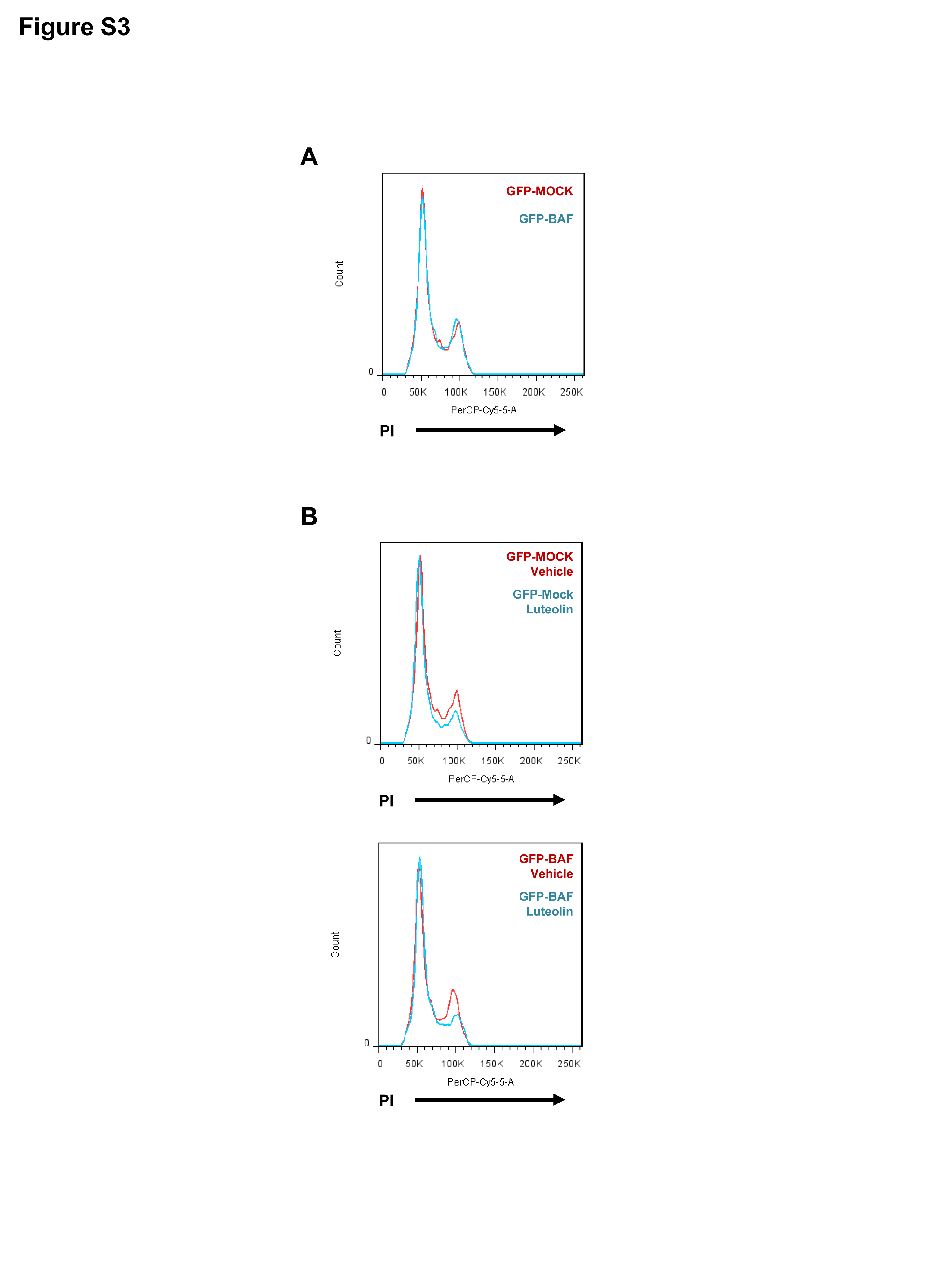

Supplement: Figure S3 — Ectopic expressed BAF does not influence cell cycle distribution, and does not rescue luteolin-induced G1 phase arrest. (A) HeLa cells were transfected with GFP or GFP-BAF, and then were stained with PI for analyzing DNA contents. Cell cycle analysis was carried out by flow cytometry. (B) GFP or GFP-BAF overexpressing cells were treated with or without 10 µM luteolin for 24 hours, and then were stained with PI. Cell cycle analysis was performed by flow cytometry. (TIF) [file pone.0109655.s003.tif]

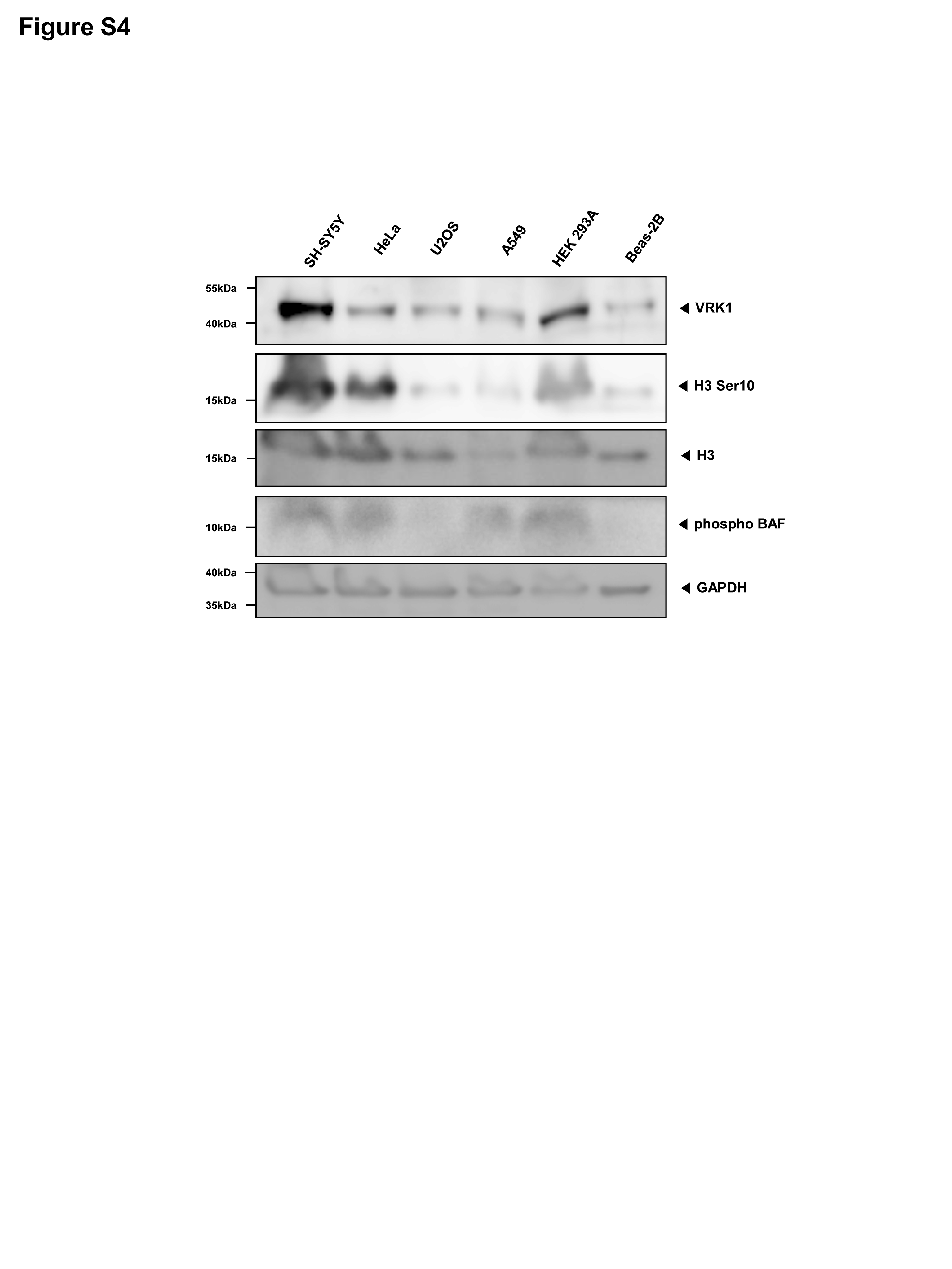

Supplement: Figure S4 — Endogenous expression levels of VRK1 and phosphorylation of BAF and Histone H3 in various cell lines (HeLa, U2OS, SH-SY5Y, BEAS-2B, HEK293A, A549). Each proteins and its phosphorylation level were detected by immunoblotting with indicated antibodies when loaded with 20 µg of each cell extracts. (TIF) [file pone.0109655.s004.tif]
